# Chronic stress accelerates the process of gastric precancerous lesions in rats

**DOI:** 10.7150/jca.52658

**Published:** 2021-05-13

**Authors:** Jiayi Zheng, Weiwu Cai, Xuen Lu, Wei He, Ding Li, Haoyu Zhong, Liangjun Yang, Siyi Li, Haishan Li, Sereen Rafee, Ziming Zhao, Qi Wang, Huafeng Pan

**Affiliations:** 1 Science and Technology Innovation Center, Guangzhou University of Chinese Medicine, Guangzhou, China.; 2 Institute of Gastroenterology, Guangzhou University of Chinese Medicine, China.; 3 Clinical Medical College of Acupuncture Moxibustion and Rehabilitation, Guangzhou University of Chinese Medicine, Guangzhou, China.; 4 Institute of Clinical Pharmacology, Guangzhou University of Chinese Medicine, Guangzhou, China.; 5 The First Affiliated Hospital of Guangzhou University of Chinese Medicine, Guangzhou, China.; 6 Second Clinical Medical College of Guangzhou university of Chinese Medicine.; 7 Rutgers University Graduate School of Biomedical Sciences, Newark, NJ, USA.; 8 Guangdong Province Engineering Technology Research Institute of Traditional Chinese Medicine, Guangzhou, China.

**Keywords:** gastric precancerous lesions (GPL), chronic stress, animal model, depression

## Abstract

**Background:** Gastrointestinal cancers account for 20% of all deaths worldwide. Gastric cancer (GC) patients are susceptible to psychological change, especially depression which is commonly induced by chronic stress. Gastric precancerous lesions (GPL) is an important prodromal stage in the occurrence of gastric cancer. Chronic stress influences the prognosis of GC and may influence the process of GPL as well.

**Methods:** Sixty SD rats were randomly divided into a control group, GPL group, and GPL+CUMS group. In the GPL group, 200μg/mL N-methyl-N'-nitro-N-nitrosoguanidine (MNNG) free drinking method combined with intermittent fasting was applied to establish the GPL animal model. Based on this, we combined the GPL rats with chronic unpredicted mild stress (CUMS) to establish a comprehensive model. We then evaluated their behavior by open field tests and sucrose preference tests. We tested the IL-6, IL-10, TNF-α, Ghrelin, Leptin and Somatostatin (SS) levels in serum and observed the expression of Ghrelin and Gastrokine 2(GKN2) in the gastric mucosa of rats with tumors by immunofluorescence.

**Results:** Our results showed that GPL and GPL+CUMS rats all displayed a significantly decreased total distance and mean velocity traveled in the open field test. The percentages of sucrose preference were significantly decreased in the GPL+CUMS group compared to the control group. In addition, IL-6 and TNF-α were significantly increased in both the GPL and GPL+CUMS groups. Furthermore, the GPL+CUMS group showed significantly increased TNF-α levels in serum compared to the GPL rats. Our results showed that the expression of NF-κB, p53, and BCL-2 were significantly increased while BAX was reduced in the GPL and GPL+CUMS groups. Moreover, Ghrelin and Leptin levels in serum were significantly decreased in the GPL and GPL+CUMS groups. SS levels in serum were significantly increased in the GPL+CUMS group. Additionally, we found that the GPL+CUMS rats with tumors not only had strong expression of GKN2 on the luminal side and the lamina propria of the gastric mucosa and tumor, but also had expression of Ghrelin on the luminal side of the gastric mucosa. The areas that showed strong expression of GKN2 and Ghrelin, are all located around the blood vessels in the tumor.

**Conclusions:** GPL rats under chronic stress would aggravate the conditions of GPL, shorten the process of GPL, and increase the risk of tumorigenesis. In addition, the close monitoring of the mental health of cancer survivors and precancerous lesion patients is suggested to be of great significance in the prevention and treatment of cancer.

## Introduction

Gastric precancerous lesions (GPL) serve as a critical stage in the development of chronic atrophic gastritis (CAG) to gastric cancer (GC) or gastric adenocarcinoma 1, 2. Slowing the process of precancerous lesions plays an important role in reducing the incidence of gastric cancer and prolongs the life of cancer survivors. Previous studies have shown that depressive disorder is highly prevalent in patients with gastrointestinal diseases 3. Gastrointestinal departments in general hospitals have investigated this matter and their results showed that nearly half of the chronic gastritis patients suffered from psychological problems such as anxiety and depression 3, 4. Some studies discovered inflammation in the stomach would cause anxiety-like and depression-like behaviors in experimental animals through neuroendocrine pathways 5, 6. Thus, depression may be frequently associated with gastrointestinal inflammations. Depression is a mental health disorder which varies from mild to severe changes in mood and affects physical, mental, and behavioral health. Chronic stress, environmental factors, socioeconomic conditions, lifestyles, and eating habits are closely related to the formation of long-term depression. Similarly, depression would influence the condition of diseases. Depression is at least two to three times more common in people with gastrointestinal disorders (GI) than in the general population, by some estimates 7. Some researchers found around 50% of investigated patients with GC suffered from depression and their life quality decreased with aggrandizing anxiety and depression 8, 9. Therefore, more attention should be paid to the mental health of the gastrointestinal disease patients.

Chronic inflammatory processes, which are associated with stress might be a fundamental symptom of depression, especially in cancer patients 10. However, the influences of depression were rarely of concern in GPL. Based on our previous studies 1, 11, we explored the pathologic structure and ultrastructure of stomach mucosal disease in GPL rats, which applied N-methyl-N'-nitro-N-nitrosoguanidine (MNNG) free drinking method combined with intermittent fasting. Consistent with Correa's findings 12, we found chronic atrophic gastritis, specialized intestinal metaplasia (SIM), cardia intestinal metaplasia (CIM), and dysplasia (Dys) appeared in the GPL group successively. We considered that the morphological abnormality, like intestinal metaplasia (IM) and dysplasia, incorporated inflammatory within gastric mucosa, may be the histological basis of gastric cancer 13-17. Some studies showed that inflammation had close relationship with depression in some diseases 18-20.

In this study, we compared two kinds of GPL animal models. One uses the GPL animal model we used in a previous study while the other uses a combination of GPL with chronic unpredicted mild stress (CUMS) to establish a comprehensive GPL model 21-24, which would be similar to clinical patients. In order to find out whether GPL rats would suffer from depression and whether depression influences the process of GPL and food intake, we administrated a dynamic observation to compare the difference of histopathology and food consumption alteration, as well as observed the inflammation factors and gastrointestinal hormone change at the final stage. Inflammation is closely associated with tumorigenesis, progression, and metastasis. We hypothesis that chronic stress would increase the inflammation, impact the gastrointestinal hormone secretion, the cell proliferation and apoptosis, involve in feeding function and appetite alteration, and result in accelerating the process of gastric precancerous lesions in rats.

## Materials and Methods

### Subjects

Sixty Sprague Dawley (SD) male rats (aged 4-6 weeks, 100-140g) were provided by the Animal Medical Center of Sun Yat-sen University (Experimental animal production license number: SCXK, Guangdong, NO.2016-0029). All the rats were raised and all animal experiments were carried out in the specific pathogen-free (SPF) Animal Medical Center of Guangzhou University of Chinese medicine, at the room temperature (18-25 °C) in 60-80% humidity (Environmental facility license number: SYXK, Guangdong, NO. 2010-0059). We measured the weight of rats before and at the 8^th^, 12^th^, 16^th^, 20^th^, 24th and 28^th^ week. Daily food consumption was measured every day at around 10-12 am. Daily food consumption = Total food weight - remnant food weight.

### GPL procedure

All animals' procedures were approved by the Committee of Anima Use for Research at Guangzhou University of Chinese Medicine (China). Sixty SD male rats were divided into a control group, GPL group and GPL+CUMS group. The control rats were given normal diet. The GPL rats were treated with 200 μg/mL N-methyl-N'-nitro-N-nitrosoguanidine (MNNG, TGI, Japan, lot number: NJAII-CK) solution and intermittent fasting (24 h of fasting every other day).

### CUMS procedure

The GPL+CUMS group was combined with chronic unpredicted mild stress (CUMS), based on the animal model of GPL. The establishment method of CUMS refer to the previous studies 21-24, with a slight modification. GPL+CUMS rats would receive two different stress stimulations every day. The same kind of stimulation didn't occur continuously so that the animal would not expect the occurrence of stimulation. The stressors consisted of the following: (A) irregular day and night inversion living; (B) 24 hours in a wet cage; (C) 24 hours in a cage tilted at 45◦ from the horizontal; (D) 24 hours of social crowding; (E) 24 hours of fasting, which was synchronized to GPL group; (F) intense light exposure for 24 hours; (G) administrated 30 V electric foot shock every 5 second for 2 minutes; (H) binding for 10 minutes.

### Open-field behavior test

The open-field behavior test was measured one day before the rats were sacrificed. Each rat was housed in a 100 cm × 100 cm × 40 cm clean chamber. Then we recorded its total distance, mean speed, the time and distance in the central square among the test. We also calculated the ratio of the time and distance in the central square, compared to the total distance and time 25.

### Sucrose preference (SP) test

The whole process of the sucrose preference test was divided into four days. We gave the rats 2 bottles of 1% sucrose solution for 2 hours on the first day. On the second day, we gave them one bottle of 1% sucrose solution and one bottle of water for 2 hours. The placement of the two bottles was alternated several times to avoid any preferences. The rats fasted for 8 hours before the test. On the last day, we gave them one bottle of 1% sucrose solution and one bottle of water for 2 hours. The weight of the bottles was recorded before and after the test. Subsequently, we analyzed the consumption of sucrose and water and calculated the SP (sucrose consumption/total intake × 100%) 24, 26, 27.

### Tissue preparation

To dynamically observe the pathological changes, three rats were randomly selected from the GPL and the GPL+CUMS groups, and were sacrificed after the 12^th^, 16^th^, 20^th^ and 24^th^ week. After 28 weeks, all the rats fasted for 8 hours but were allowed to drink and were anesthetized by Pentobarbital sodium (0.2 ml/100g, i.p.) before they were sacrificed. Blood samples were collected from the abdominal aorta. The blood samples were centrifuged at 3000 r/min for 15 minutes to collect the supernatant which was stored in the refrigerator at -80 °C. The gastric tissue was removed from the abdomen and cut along the greater curvature of stomach. The stomach contents were removed and rinsed with PBS (4 °C). The gastric tissue was unfolded to observe the damage of the gastric mucosa and fixed in 4% paraformaldehyde at 4 °C. After dehydrated by and embedded in paraffin, the gastric tissues were sliced into 4 μm thick sections.

### Enzyme-linked immunosorbent assay (ELISA)

We used rat interleukin (IL)-10 Elisa Kit (Jianglai Biological, China, JL13427), rat IL-6 Elisa Kit (Jianglai Biological, China, JL20896), rat tumor necrosis factor-α Elisa Kit (TNF-α) (Jianglai Biological, China, JL10707), rat Ghrelin Elisa Kit (Jianglai Biological, China, JL21321), rat Leptin Elisa Kit (Jianglai Biological, China, JL18311) and rat SS Elisa Kit (Jianglai Biological, China, JL12919) to assess the expression levels in serum. We followed the instructions to administer the tests. The optical density value (OD value) of each well was measured by a microplate reader at a wavelength of 450 nm and the measurement process was carried out within 15 minutes of the addition of the stop solution 28.

### Histopathological Observation

The sections of gastric tissues would stain with Hematoxylin and Eosin (H&E, Leagene, DH0006), Alcian blue/Periodic acid Schiff (AB/PAS, BASO, BA4121), High iron diamine/Periodic acid Schiff (HID/AB, BASO, BA4120A) dye 29. The sections were placed on the slide warmers at 60 °C to melt the paraffin. The sections were then stained with Hematoxylin and Eosin (HE), Alcian blue/Periodic acid Schiff (AB/PAS) and High iron diamine/ Alcian blue (HID/AB). Further, the sections were rehydrated, dehydrated and sealed the tissue conventionally. All sections were observed under microscope.

### Immunofluorescence

The sections of gastric tissues were placed on the slide warmers at 60 °C to melt the paraffin. After rehydrated, the sections were immersed into an antigen retrieval solution (1X) (Beyotime Biotechnology, P0088) and heated in water bath at 95-100 °C for 20 minutes. We used a permeabilization buffer (Beyotime Biotechnology, P0097-500M) for 5 minutes and a blocking buffer (Beyotime Biotechnology, P0102) for 1 hour at room temperature. The sections were incubated with antibodies against Ghrelin (1:100, Abcam, AB209790) and Gastrokine 2 (GKN2, 1:1000, Abcam, AB188866) at 4 °C overnight. The sections were then incubated with Alexa Fluor 488-labeled Goat Anti-Rabbit IgG (H+L) (Beyotime, 1:500; A0423) secondary antibodies for 1 hour at room temperature in the dark. They were sealed with anti-fade mounting medium with DAPI (Beyotime, 1:500; P0131). All sections were observed under fluorescence microscope.

### Western blotting

The gastric tissues were homogenized, lysed, and centrifuged at 12,000 rpm for 30 min at 4 °C. The protein concentrations were quantified using the BCA kit. The samples were then denatured by boiling at 100 °C with loading buffer. Equal amounts of proteins were fractionated by SDS-polyacrylamide gel electrophoresis (PAGE) and transferred onto PVDF membrane. The membrane was blocked with 5% skim milk at room temperature for 1 hour and then incubated with anti-Bcl-2(Abcam, ab182858), anti-Bax (CST, #5023), anti-p53 (Abcam, ab26), anti-NF-κB p65 (Abcam, ab16502) and anti-β-actin (CST, #8457) antibody overnight at 4 °C. Anti-rabbit or anti-mouse secondary antibody was added and incubated for an hour. Super enhanced chemical luminescence reagent (ECL; Applygen Technologies Inc., Beijing, China) was used for imaging. The bands were quantified by Imagelab software.

### Statistical Analysis

Data are represented as mean ± SEM. The data presented above was processed using SPSS 19.0 statistical software. Data were analyzed with two-way repeated analysis of variance (ANOVA) or one-way ANOVA. P<0.05 was considered statistically significant.

## Results

### GPL and GPL+CUMS could induce loss of weight

**Figure 1:**
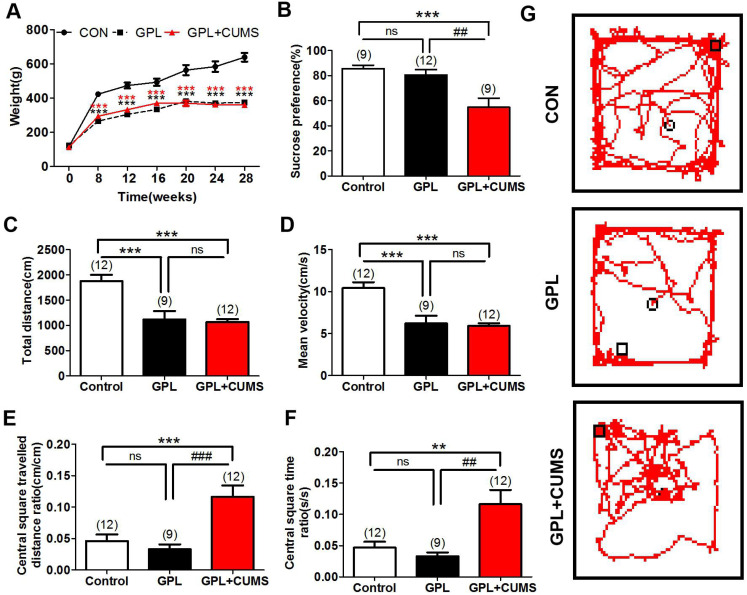
(A) Weight alteration; (B) Sucrose preference test; (C) Open-field behavior test analysis: Total distance; (D) Open-field behavior test analysis: Total Mean velocity; (E) Open-field behavior test analysis: Total Central square travelled distance ratio; (F) Open-field behavior test analysis: Total Central square time ratio; (G) Open-field behavior test analysis: Total Track route (*P < 0.05, **P < 0.01, ***P < 0.001, compared to Control group; ## P < 0.01, ### P < 0.001, compared to GPL group. Data are represented as mean ± SEM).

As we know, chronic diseases may lead to insufficient energy supply in the body and weight loss. In our study, there was no significant difference in body weight among the three groups before modeling (P >0.05, Fig. 1A). The weight of the rats in the three groups increased rapidly at first and then showed a slow upward trend. The weight of three groups had a significant increase at the 8^th^, 12^th^, 16^th^, 20^th^, 24^th^ and 28^th^ week compared to their weight before modeling (*P* <0.001, Fig. 1A). We found that the GPL and GPL+CUMS rats have a significant delay in their weight gain. Compared to the weight of age-matched control rats, there was a significant difference in the GPL rats and the GPL+CUMS rats at the 8^th^, 12^th^, 16^th^, 20^th^, 24^th^ and 28^th^ week (*P* <0.001, Fig. 1A). After the 20^th^ week, the development of weight began to stabilize in the GPL and GPL+CUMS rats. However, the alteration of weight had no significant difference between the GPL and GPL+CUMS groups, regardless of whether it is before and after modeling (P >0.05, Fig. 1A). The development of GPL is based on CAG. Aggravation of CAG and GPL results in weight loss.

### GPL+CUMS could influence the behavior

Among many cancer patients, depression is a common symptom 30 that serves as a potential indication of aggravation and poor prognosis. Chronic stress plays an important role in depression since stress increases the risk for its occurrence and development 31. It is associated with increased unexpected stressful events, feelings of loneliness, and unhealthy conditions.

To investigate whether GPL rats have behavior alterations, we evaluated the behaviors utilizing an open field test among three groups. The resulted showed that GPL and GPL+CUMS rats all displayed a significantly decreased total distance (P< 0.001, Fig. 1C) and mean velocity (P < 0.001, Fig. 1D) traveled in the whole open field. This means that the general locomotion of GPL and GPL+CUMS rats was decreased. However, the ratio of distance travelled in the central square and the total distance of GPL+CUMS rats were significantly increased, compared to the control (P < 0.001) and GPL rats (P < 0.001, Fig. 1E, 1G). In addition, the ratio of the time spent in the central square and the total time of the GPL+CUMS rats were significantly increased, compared to the control and GPL groups (P < 0.01, P < 0.01, Fig. 1F). This data indicates that GPL+CUMS rats had lower motivation for exploration and preferred to stay in one quadrant area of the open field, expressing depression-like behaviors.

### Chronic stress contributed to depression in GPL rats

Chronic stress induces several behavioral changes. Anhedonia is considered as depressive-like behavior 32. To explore whether GPL rats with chronic stress experienced depression, we used sucrose preference to assess depressive-like behaviors. Our results showed that the percentages of sucrose preference were significantly decreased in GPL+CUMS group (P < 0.001, P < 0.001, Fig. 1B), compared to the control and GPL+CUMS groups. It proved that chronic stress contributed to depression.

### GPL+CUMS rats had more significant appetite alteration

**Figure 2:**
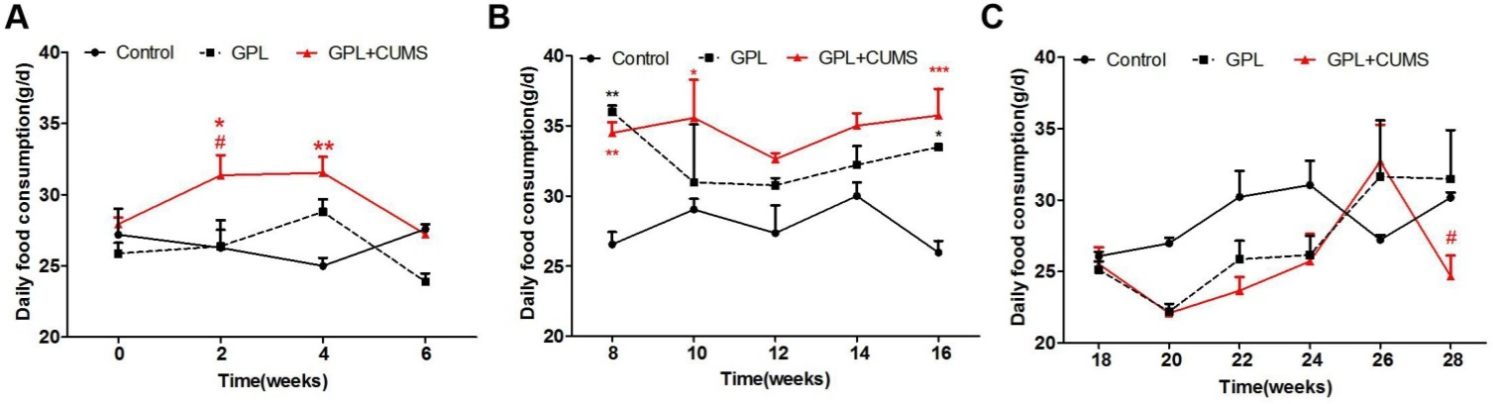
(A) Daily food consumption (From 0^th^ week to 6^th^ week); (B) Daily food consumption (From 8^th^ week to 16^th^ week); (C) Daily food consumption (From 18^th^ week to 28^th^ week) (*P < 0.05, **P < 0.01, ***P < 0.001, compared to Control group; # P < 0.05 compared to GPL group. Data are represented as mean ± SEM).

Compared to age-matched control rats, the GPL and GPL+CUMS rats had a wider range of daily food consumption. The daily food consumption of the GPL+CUMS rats changed as early as the 2^nd^ week (Fig. 2A). From the 8^th^ week on, the daily food consumption of the GPL rats and the GPL+CUMS rats showed a large upward trend and consumed more food than the control rats from the 8^th^ to 16^th^ week, which may be induced by the intermittent diet (Fig. 2B). The daily food consumption showed a large downward trend from the 16^th^ week and then increased slowly. From the 26^th^ week to the 28^th^ week, the daily food consumption of the GPL+CUMS rats dropped significantly (Fig. 2C). These results showed that the GPL and GPL+CUMS rats had an increase in daily food consumption in the early stage, which is similar to binge eating after fasting. In the later period, the GPL and GPL+CUMS rats had a decrease in food intake under an unstable state, which is similar to appetite alteration. GPL+CUMS rats had more significant change in appetite alteration.

### GPL+CUMS rats could accelerate the development process of GPL

**Figure 3:**
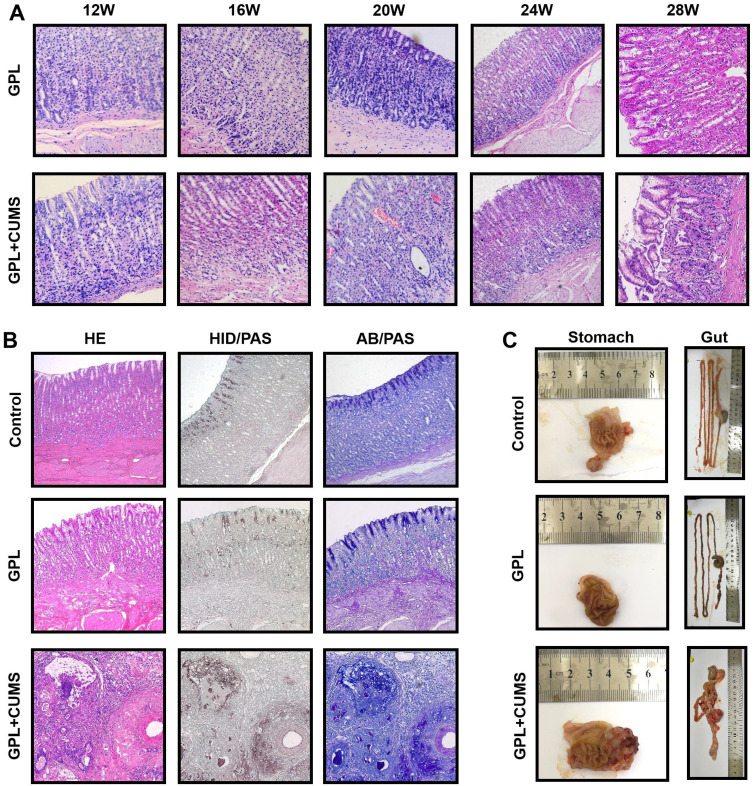
(A) 12^th^, 16^th^, 20^th^, 24^th^ and 28^th^ week pathology observation of GPL and GPL+CUMS group (stomach; ×200, HE). (B) 28^th^ week pathology observation of Control, GPL and GPL+CUMS group (stomach; ×100: HE, HID/PAS, AB/PAS); (C) Macroscopic appearance of Control, GPL and GPL+CUMS group (stomach and gut).

The histopathological characteristics of GPL are based on CAG, with moderate and severe dysplasia and with or without incomplete colonic metaplasia. GPL is an important and prodromal stage of cancer. In order to find out whether chronic stress aggravates the development of GPL, we reported chronological changes of gastric mucosa in the GPL and GPL+CUMS groups (Fig. 3A). In the 12^th^ week (Fig. 3A), gastric mucosa displayed an inflammatory response consisting of infiltration of small round cells, mainly lymphocytes (Fig. 3A) and cystic dilation occurred in gastric glands in both groups. The gastric glands started to reduce and display vacuolation as early as the 12^th^ week in the GPL+CUMS group, while the GPL group started in 16^th^ week. In the 16^th^ week, we found regenerative thickening in the muscular layer, as well as early vascular lesions and increased number of irregular arrangements in gastric glands in both groups, but it was more prominent in the GPL+CUMS group. The GPL+CUMS group showed abundant capillaries as early as the 20^th^ week, while the GPL group did in the 24^th^ week. Dysplasia (Dys) and intestinal metaplasia (IM) changes were observed in the 24^th^ week in the GPL+CUMS group, while the GPL group was in the 24^th^ week. IM developed from the luminal side of the digestive tract. The gland ducts advance deeply and gradually followed by cystic proliferation that occupies the mucosa 33. In the 28^th^ week, we discovered that IM in some samples had occupied the whole mucosal layer, replacing gastric glands.

Relatively, gastric mucosa tissue in the control group showed intact appearance of gastric glands (Fig. 3B). In our study, we only found two rats that suffered from gastric tumors at the 28^th^ week, and one of them had cancer metastasis. Both are from the GPL+CUMS group. As we expected, we found that chronic stress not only could accelerate the development process of GPL (Fig. 3A, 3B), but could also result in the appearance of tumors (Fig. 3B, 3C). We found some glandular tubes and inflammatory cells inside the tumor. Its adenocarcinoma is characterized by mucinous carcinoma, according to the AB/PAS and HID/AB stains.

### GPL+CUMS could increase inflammatory reaction and influence secretion function of gastric mucosa

**Figure 4:**
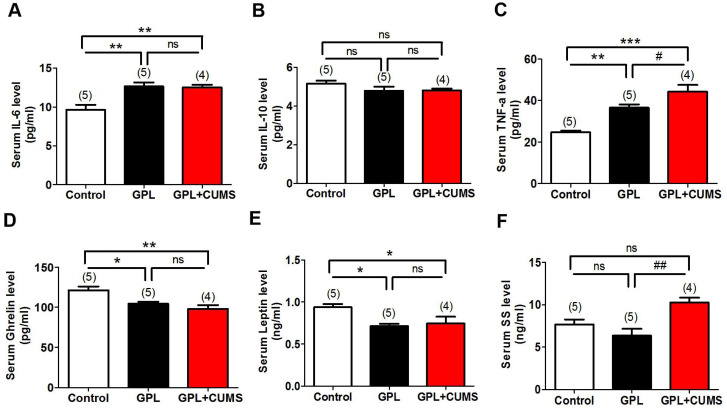
(A-F) Elisa analysis of IL-6, IL-10, TNF-α, Ghrelin, Leptin and SS levels in serum (*P < 0.05, **P < 0.01, ***P < 0.001, compared to Control group; # P < 0.05, ## P < 0.01, compared to GPL group. Data are represented as mean ± SEM).

Inflammation plays an important role in the development of disease, including cancers. To explore whether Inflammation is involved in the development process of GPL and find out whether chronic stress accelerates the process of GPL by increasing inflammation, we measured IL-6, IL-10 and TNF-α levels in serum among three groups. Our results showed that serum IL-6 level was significantly increased in the GPL and GPL+CUMS groups compared to control group (P < 0.01, Fig. 4A). There was no difference between the GPL and GPL+CUMS groups (P > 0.05, Fig. 4A). TNF-α level in serum had a significant increase in the GPL and GPL+CUMS groups (P < 0.01, P < 0.001, Fig. 4C), compared to control group. Moreover, GPL+CUMS showed a significantly increased TNF-α level in serum compared with GPL rats. As for serum IL-10 level, there was no difference among the three groups (P > 0.05, Fig. 4B). This means inflammation involved in the development process of GPL and chronic stress could increase inflammatory cytokines such as TNF-α and IL-6.

In addition, Ghrelin is one of the peptides produced by the gastric mucosa. Under conditions of chronic inflammation and atrophy in the process of GPL, the concentrations of peptides which were mainly produced by gastric mucosa in serum would decrease 34. Ghrelin and Leptin are closely related to food and energy consumption. Based on our results, Ghrelin and Leptin levels in serum were significantly decreased in the GPL and GPL+CUMS groups, compared to the control, while the increased expression of Ghrelin was more obvious in the GPL+CUMS group (P < 0.05, Fig. 4D, 4E). Compared to the control and GPL rats, serum SS level was significantly increased in the GPL+CUMS group (P < 0.01, Fig. 4F).

Taken together, the inflammation in gastric mucosa may be the histological basis of GPL. TNF-α, IL-6 and Ghrelin in serum might be the target to assess the degree of GPL.

### Chronic stress could increase cell proliferation and decrease apoptosis, contributed to the development of GPL

**Figure 5:**
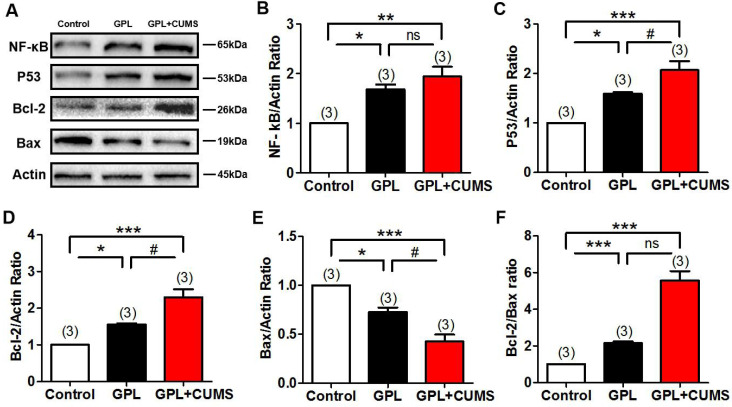
(A)The expression of NF-κB, P53, Bcl-2, Bax and β-Actin in the gastric tissues; (B) NF-κB/Actin ratio; (C) P53/Actin ratio; (D) Bcl-2/Actin ratio; (E) Bax/Actin ratio; (F) Bcl-2/bax ratio (*P < 0.05, **P < 0.01, ***P < 0.001, compared to Control group; # P < 0.05 compared to GPL group. Data are represented as mean ± SEM).

To explore whether chronic stress accelerates the process of GPL, we measured the expression of NF-κB, P53, Bcl-2 and Bax in the gastric tissues, which are the associated biomarker of inflammation, proliferation, apoptosis and malignancy. NF-κB system is a key in linking inflammation and cancer 35. Our results showed that NF-κB was significantly increased in the GPL and GPL+CUMS groups, compared to the control group (P < 0.05, P < 0.01, Fig. 5A, 5B). The overexpression of P53 protein is closely related to the occurrence of gastric cancer and plays an important role in the development of GPL and GC. The expression of P53 had a significant increase in the GPL and GPL+CUMS groups (P < 0.05, P < 0.001, Fig. 5A, 5C), compared to the control group. Further, GPL+CUMS showed a significantly increased expression of P53 compared to the GPL rats.

Bcl-2 and Bax are the specific biomarkers of proliferation and apoptosis. Our results showed that the expression of Bcl-2 was significantly increased and Bax was significantly decreased in the GPL and GPL+CUMS groups (P < 0.05, P < 0.001, Fig. 5A, 5D, 5E), compared to the control group. Compared to GPL group, the expression of Bcl-2 and Bax both had significant change in the GPL+CUMS group (P < 0.05, P < 0.05, Fig. 5A, 5D, 5E). Bcl-2/Bax ratio was significantly increased in the GPL and GPL+CUMS groups, compared to the control group (P < 0.001, P < 0.001, Fig. 5F). This means inflammation involved in the development process of GPL and chronic stress could increase cell proliferation and decrease apoptosis which would contribute to the development of GPL.

### GKN2 and Ghrelin might serve as the potential roles in the process of GPL

**Figure 6:**
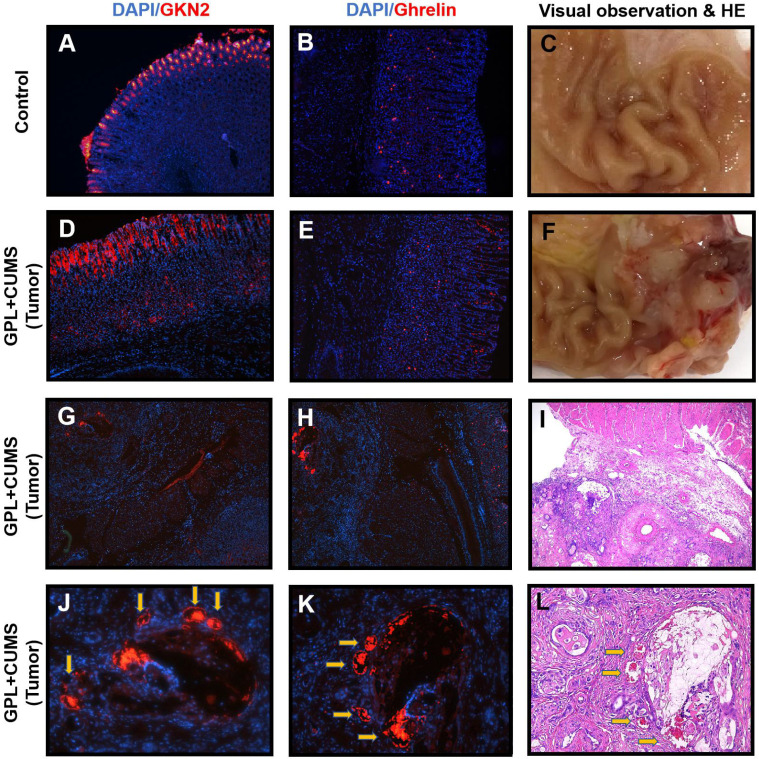
(A) Immunostaining of GKN2 in the gastric tissues of Control rat (28^th^ week, ×100); (B) Immunostaining of Ghrelin in the gastric tissues of Control rat (28^th^ week, ×100); (C, F) Macroscopic appearance of the gastric tissues of Control rat and GPL+CUMS rat (28^th^ week); (D, G, J) Immunostaining of GKN2 in the gastric tissues of GPL+CUMS rat with tumors (28^th^ week, ×100, ×50, ×200); (E, H, K) Immunostaining of Ghrelin in the gastric tissues of GPL+CUMS rat with tumors (28^th^ week, ×100, ×50, ×200); (I, L) Pathology observation of the gastric tissues of GPL+CUMS rat with tumors (28^th^ week, HE, ×50, ×100). GKN2 (red) and Ghrelin (red) were detected using with specific antibodies. Cell nuclei in the sections were stained with DAPI (Blue).

In our experiments, we found that two rats suffer from tumors (Fig. 6F, 6I). We used immunofluorescence to observe the expression of Ghrelin and GKN2 in gastric tissues near and within the tumors.

In order to better evaluate the indicators of gastric mucosal function, this study observed the expression of GKN2 in gastric mucosa. GKN2 is expressed in normal gastric mucosal cells (Fig. 6A). We found a GPL+CUMS rat with tumors that had a strong expression of GKN2 on the luminal side of gastric mucosa, which may be related to the degree of intestinal metaplasia. We also found GKN2 expressed in lamina propria of gastric mucosa and tumor of the rat suffered from tumors (Fig. 6D, 6E, 6J). Interestingly, compared to the HE stains, the places that had strong expression of GKN2 and Ghrelin, were located around the blood vessels in the tumor (Fig. 6J, 6K, 6I).

Due to the reduction of gastric glands in the GPL process, the level of Ghrelin concentration in whole body decreases. This is proven in previous results. Based on our findings, we observed that Ghrelin is produced in the fundic glands of the stomach. We found Ghrelin mostly expressed in lamina propria of gastric mucosa of control rat (Fig. 6B). However, the GPL+CUMS rat that suffered from tumors had the expression of Ghrelin on the luminal side of gastric mucosa (Fig. 6E).

## Discussion

Chronic stress is a major harmful factor in our society as it elicits symptoms like depression and anxiety. Comorbid diseases are the important issues in cancer research 36. However, many people do not keep a watchful eye on the mental health of cancer survivors and the patients with precancerous lesions. In the clinical observation, patients with gastrointestinal diseases may be prone to suffer from psychologic changes, such as anxiety, depression and etc. On the other hand, patients with encephalopathy or psychological illnesses may have different degrees of gastrointestinal dysfunction and appetite alteration. Therefore, depression can be one of the potential factors of poor prognosis in gastric cancer patients 33. Stress and depression are related to many gastrointestinal diseases and depression may also be a precipitating factor of gastric cancer 37. Chronic stress is a widely acknowledged as a predisposing factor of depression. Extensive research shows chronic stress induces several behavioral alterations such as depressive-like behavior. During the development of CAG, GPL to gastric cancer, clinical gastrointestinal function and appetite changes could be easier to figure out, compared to psychological condition changes, such as depression-like behavior.

To find out whether GPL rats show behavior alteration, we applied MNNG free drinking combined with intermittent fasting to establish a GPL model, and observed their behaviors. MNNG, a chemical mutagenesis agent and carcinogen, is currently recognized as a common drug to widely and effectively establish CAG, GPL and GC models in previous studies. Long-term intake of MNNG can easily induced CAG, and eventually form gastric cancer. According to the results of open field, GPL presented less general locomotion and preferred to stay along the sides of the field, showing depression-like behaviors. To further confirm whether they suffer from depression, we assess by sucrose preference test. GPL rats did not show the loss of reward pleasure, which indicated they did not suffer from depression. We consider that long-term illness might lead to insufficient energy supply in the body, which may be displayed by weakened motility. However, it may also be caused by other factors, such as pain and other uncomfortable conditions induced by GPL. To further explore whether chronic stress takes part in the development of GPL, we applied GPL rats with CUMS to imitate the GPL patients under chronic stress. Depending on the results of the open field, GPL+CUMS rats presented less general locomotion as GPL rats. Interestingly, the GPL+CUMS rats prefer to stay in one quadrant of field. Under chronic stress, GPL+CUMS rats feel lonelier and down, and experience loss of curiosity to explore the environment. All control rats showed increased curiosity and activity. As expected, GPL rats under chronic stress lose the pleasure of reward and induce depression-like behaviors.

We administrated a dynamic observation to further confirm the influence of chronic stress in GPL rats. Histopathological diagnosis is the golden standard. Through dynamic observation, we found the GPL and GPL+CUMS rats showed different histopathology among 28 weeks. In the beginning, they all presented the histopathological characteristics of CAG, for example, atrophy of the gland in the stomach. The degree of atrophy is calculated by the reduction of intrinsic glands among the stomach. When GPL+CUMS rats appeared the moderate degree of atrophy in the 12^th^ week, GPL rats stayed on the mild degree. In the 16^th^ week, some of GPL rats under chronic stress appeared to have severe atrophy, while GPL rats remained on the mild and moderate degree. In addition, Dys and IM are important assessment points of histopathology. IM might act as an alternative marker, to evaluate the risk of GC and malignant potential 38. We observed GPL rats occurred Dys and mild intestinal metaplasia in the 24^th^ week, but GPL+CUMS rats appeared one month earlier and it reached the sever degree in the 28^th^ week. The injury of capillaries and angiogenesis are special indicators in GPL. In the early stage of GPL, the capillaries in gastric mucosa were high-density, while the density of capillaries would drop off by degrees. In the late stage of GPL, angiogenesis would appear. We found early vascular lesions in the 16^th^ week in the GPL and GPL+CUMS groups, but those in the GPL+CUMS group are more prominent. Furthermore, the GPL+CUMS group possessed abundant capillaries in the 20^th^ week, one month earlier than the GPL group. Interestingly, the more serious lesion area had more abundant capillaries. Some researchers found that in the area of gastric cancer, the overall incidence of genetic instability (GIN) would have a significant increase with lymphatic or vascular invasion 39. In our previous studies, we hypothesized that the blood metastasis might happen before the cancer cells infiltrating the basement membrane in GPL. Based on our previous researches, we believed abundant capillaries might evolve into neovascular cancer in the future. Moreover, with the accumulation of long-term stress the process of GPL became faster after 24 weeks. The two rats that suffered from gastric tumors in our study could be of evidence. All in all, our findings proved that under chronic stress stimulation, the process of GPL is increased which may easily lead to tumorigenesis. Some studies indicated that metaplasia appears to be an adaptive response 33 and acts as self-protection. Chronic inflammation may also be an important factor.

Thus, we assessed the inflammatory reaction among three groups to investigate whether depression influences the development of GPL by mediating inflammation. In our study, gastric mucosa had caused an inflammatory response around the 12^th^ week and it continued throughout the whole process. Combined with the results of the ELISA tests, we found that the concentration of IL-6 and TNF-α, as the inflammatory cytokines, showed significant changes in serum, while the post-inflammatory cytokines, like IL-10, showed no significant changes among the three groups. In this study, higher TNF-α and IL-6 levels reflected the inflammation in the whole body. TNF-α and IL-6 may be significant and observable targets. Somatostatin (SS), is an important gastrointestinal hormone which could inhibit gastrin secretion 40. In our studies, SS levels expression in serum were significantly increased in the GPL+CUMS group which is a key target during the process of CAG and GPL. Inflammation is an important factor in GPL. Studies have shown that NF-κB may facilitate tumorigenesis by regulating inflammation, cell proliferation, and apoptosis. P65 is the most pivotal subunit of NF-ΚB and the activation of NF-κB contributes to GPL 41. Our results showed that chronic stress significantly increases the expression of p65 in GPL rats.

Inflammation contributes to the tumorigenesis, progression, and metastasis. P53 is a major tumor suppressor, which serves a major role in metabolism, regardless of whether it is in normal or cancer cells 42, 43. The increasing dysregulation of p53 would increase the risk of gastric cancer 44. In our previous studies, we had reported that p53 proteins were up-regulated in GPL rats 1. In this research, we found that chronic stress significantly increased the expression of p53. Moreover, Bcl-2 and Bax are two major proliferation- and apoptosis-related factors. Bcl-2 inhibits the cell's apoptosis, while Bax antagonistically stimulates it 45-47. In our previous studies, we found Bcl-2/Bax ratio was abnormal in GPL rats and suggested that mutation of activating p53 depends on autophagy dysfunction. From our newfound results, the expression of Bcl-2 was significantly increased and Bax was significantly decreased in both the GPL and GPL+CUMS groups. As expected, the Bcl-2/Bax ratio significantly increased in GPL+CUMS rats. All data considered, our findings suggest that chronic stress increases the inflammation and the risk of tumorigenesis as well as influence the cell proliferation and apoptosis in GPL rats.

As we know, the most typical lesion of CAG and GPL gastric mucosa is atrophy. The atrophy of gastric mucosa leads to a corresponding decrease in the secretion of hormones in mucosal cells. Inflammation may influence its production and secretion. Therefore, we assessed the concentration of the gastrointestinal hormone, Ghrelin, in serum. Ghrelin acts as an indicator to assess the function of glands because it is mainly secreted by the gastric mucosa 48. During the entire modeling period, the GPL and GPL+CUMS rats had binge eating and intermittent anorexia, of which the change in GPL+CUMS rats is more obvious. Therefore, feeding stability is an important part of gastric mucosa self-regulation and protection.

Ghrelin can affect appetite, energy balance, gastric motility, mediate inflammation and gastric acid secretion 48. However, the relationship among Ghrelin, GPL, and GPL with depression has not been investigated. Previous studies indicated that Ghrelin is closely related to upper gastrointestinal diseases 49. Inflammation and atrophic changes associated with infection may result in impaired biosynthesis of Ghrelin, resulting in decreased blood levels of Ghrelin 50. While Ghrelin has anti-inflammatory and anti-apoptotic functions, altered expression of Ghrelin can reflect the degree of gastric inflammation or the degree of gastric atrophy to some extent 51, contributed to judge the severity of the disease. Some researchers suggested the concentrations of serum Ghrelin were associated with the risk of gastric non-cardia adenocarcinoma (GNCA) and esophagogastric junctional adenocarcinoma (EGJA) 34. Ghrelin also plays a key role in mood disorders 52. Some studies found chronic peripheral administration of Ghrelin could alleviate depression-like behaviors induced by CUMS, and indicated Ghrelin had antidepressant effect 52. In this study, serum Ghrelin was significantly lower in the GPL+CUMS rats than in the GPL rats. However, we found overexpression in the gastric mucosa of GPL+CUMS rats. We suggested that may be the result of a compensatory response in GPL, to remedy for the deficiency of secretion induced by atrophy. However, the effect of Ghrelin in GPL is still uncertain. Ghrelin may play different role under physiologic or pathologic conditions. Ghrelin could be secreted by organs such as the stomach, small and large intestines, kidney, brain, the lungs and so on. Depression might influence inflammatory reactions that aggravate the development of GPL by altering the production, secretion and bond of Ghrelin and its receptors. Ghrelin and GKN2 may have different functions under physiological and pathological conditions. Gastric mucosa is the main point of GPL. Menheniott indicated frequently loss of GKN2 expression is related to the pathogenesis of GC 53. In some studies, Ghrelin and GKN2 might have anti-inflammatory effect 53, which were tumor suppressor genes. Our research suggests that the inflammation in gastric mucosa during GPL stage, might result in the low expression of Ghrelin and GKN2. However, GPL might increase the expression in blood vessels vicariously to compensate for the inadequate secretion of gastric mucosa. GKN2 and Ghrelin might serve potential roles in GPL, as well as markers of neovascularization in tumors.

Based on our findings, the aggravation of GPL could lead to weight loss. We considered that inflammation could mediate the gastrointestinal hormone secretion in GPL, which might influence feeding function and appetite alteration, and result in weight loss. The mechanisms of GPL are still uncertain. As for the treatment of GPL, there are no specific medications or treatments for it. Thus, reducing the risk factor seems to be more critical in the process of GPL. We suggested that GPL would increase inflammatory cytokines and it would be more intense under chronic stress, accelerating the progress of GPL to GC. Chronic persistent inflammatory reaction in the GPL may damage the body's normal immune function. That is one of the most important factors affecting the occurrence and development of gastric cancer. Further study is needed.

## Conclusion

Under chronic stress, rats with gastric precancerous lesions show depression-like behavior. In our studies, GPL rats under chronic stress aggravate the conditions of GPL, shorten the process of GPL, and increase the risk of tumorigenesis. Inflammation, autophagy, and the neuroendocrine system have a close relationship with the development of GPL. Chronic stress would increase the inflammation, impact the gastrointestinal hormone secretion, break the balance of the cell proliferation and apoptosis, involve in feeding function and appetite alteration, and result in accelerating the process of gastric precancerous lesions in rats. In addition, GKN2 and Ghrelin may serve potential roles in GPL, as well as the markers of neovascularization in tumors. Finally, we suggested the careful monitoring of mental health among precancerous lesion patients would be of great significance for the prevention and treatment of cancer.
